# The Motor System Contributes to Comprehension of Abstract Language

**DOI:** 10.1371/journal.pone.0075183

**Published:** 2013-09-26

**Authors:** Connie Qun Guan, Wanjin Meng, Ru Yao, Arthur M. Glenberg

**Affiliations:** 1 University of Science and Technology, Beijing, China; 2 Florida State University and Florida Center for Reading Research, Tallahassee, Florida, United States of America; 3 National Institute of Education Sciences, Beijing, China; 4 Arizona State University, Tempe, Arizona, United States of America; 5 University of Wisconsin-Madison, Madison, Wisconsin, United States of America; University of Milan, Italy

## Abstract

If language comprehension requires a sensorimotor simulation, how can abstract language be comprehended? We show that preparation to respond in an upward or downward direction affects comprehension of the abstract quantifiers “more and more” and “less and less” as indexed by an N400-like component. Conversely, the semantic content of the sentence affects the motor potential measured immediately before the upward or downward action is initiated. We propose that this bidirectional link between motor system and language arises because the motor system implements forward models that predict the sensory consequences of actions. Because the same movement (e.g., raising the arm) can have multiple forward models for different contexts, the models can make different predictions depending on whether the arm is raised, for example, to place an object or raised as a threat. Thus, different linguistic contexts invoke different forward models, and the predictions constitute different understandings of the language.

## Introduction

Common sense, as well as some formal analyses (e.g., [1], [2]), suggests that language and action are distinct mental processes. In contrast, the embodied cognition framework highlights the importance of bodily processes, such as action, for all cognition [3]. According to this framework, cognition is ‘grounded’ in sensorimotor activity (see, [4]–[12]). That is, sensorimotor processing underlies and constitutes cognition.

In the current research we investigate the sensorimotor grounding of the abstract concept of quantity, in particular, the terms “more” and “less”. Lakoff and Johnson [13] have suggested that people understand quantity using the conceptual metaphor “more is up.” If that is correct, then asking people to move their hands upward while understanding sentences consistent with “more” should be easier than asking people to move their hands downwards: an Action-sentence Compatibility Effect (ACE) [14]. We begin by reviewing the literature on sensorimotor embodiment and the ACE. We then present an experiment that uses behavioral and EEG measures to demonstrate how the understanding of quantity is grounded in sensorimotor activity. In the discussion section, we consider several reasons why the motor system is particularly important for grounding (at least some) abstract language. These reasons focus on the claims that a) prediction is central to both motor tasks and language comprehension, b) motor activity is central to the neurophysiological implementation of prediction using forward models, and c) abstract concepts are those whose meaning is greatly dependent on prediction.

### 1.1 Concrete and Abstract Concept Understanding via Sensorimotor Simulation

The embodiment framework suggests that linguistic knowledge is based on sensorimotor simulations. That is, the neural systems that are involved in perceiving and acting with objects and events in the world are also used to internally simulate those objects and events at later points in time [15]–[17]. Thus, a word such as “banana” is understood by activating perceptual and action codes acquired through experience: how bananas look, feel, taste, how to peel them, and so on.

Although embodied accounts of the sensorimotor grounding of concrete concepts are reasonably straightforward, the simulation of less tangible, abstract concepts may be less so (e.g., [18]). Nonetheless, it has been suggested that abstract concepts are similarly grounded in the body’s systems of perception and action planning (e.g., [14]–[15], [18]–[22], as well as the emotional system (e.g., [23]). We develop this idea in detail in the Discussion. Here we briefly sketch the literature on the grounding of abstract ideas.

Research suggests that sensorimotor simulations play a role in the comprehension of abstractions. For example, Glenberg and Kaschak [14] and Glenberg et al. [24] argued that the understanding of abstract transfer situations (e.g., language describing the transfer of information) is grounded in the motor system in a manner similar to the understanding of concrete transfer situations (e.g., the transfer of tangible objects). As another example, Boot and Pecher [25] found that the understanding of the concept of “categories” is grounded in the concrete representation of a container (i.e., a category is seen as a container in which some items are inside, and some are outside). Similarly, Richardson, Spivery, Barsalou, & McRae [26] found that understanding abstract verbs such as “respect” involves activation of a spatial image-schema. Kousta, et al. [23] have documented how abstract concepts, compared to concrete concepts, are more strongly grounded in emotional (i.e., interoceptive) experiences.

There are also several investigations of the question using neurophysiological techniques. For example, Aziz-Zadeh, Wilson, Rizzolatti, & Iacoboni [27] found somatotopic activation in the premotor cortex for literal action (“grasping the scissors”), but not for metaphorical usage (“grasping the idea”), and similar findings are reported by Raposo, Moss, Stamatakis, & Tyler [28] In contrast, Boulenger, Shtyrov, & Pulvermüller [29] do report somatopic activation for metaphorical usage of action verbs, as do Desai, Binder, Conant, Mano, & Seidenberg [30]. There are probably many reasons for these inconsistencies, including different languages, different measurement techniques, and different stimuli.

In our research we take a different tack. Rather than investigating the metaphorical usage of action verbs, we focus on the quantifiers “more” and “less.” Because these quantifiers do not have an obvious motor component in either their literal or metaphorical usages, they provide a stronger test of the embodiment claim that language is understood using sensorimotor simulations. Also, focusing on just these two quantifiers that can be used repeatedly in experimental material without sounding odd, we have the advantage of obtaining multiple observations of the same, or very similar, processing. Finally, we provide a novel interpretation of how abstract language interacts with the motor system based on notions of prediction and forward models [31]–[33].

Are quantifiers abstract? Certainly quantifiers are not concrete in the sense that they are not something that is directly sensed such as shape, weight, color, etc., nor do quantifiers correspond to a particular image (e.g., the image of a banana), or a particular action (e.g., running). In that sense, they are prototypically abstract. But given that, how can quantifiers be grounded in the sensorimotor system? In brief, we call on Barsalou’s [19] analysis that abstract concepts are not a simulation of a particular object, but the simulation of a process. Thus, a quantifier such as “more and more” is a simulation of increasing the number or amount of the object modified by the quantifier. Our research goal is to determine if there is evidence for such a simulation process in the motor system.

### 1.2 ACE and its Behavioral and ERP Evidence

In sentence comprehension, sensorimotor simulation is demonstrated by the *Action-sentence Compatibility Effect* (ACE). For example, Glenberg and Kaschak’s [14] participants responded that a sentence was sensible by moving the right hand from one button to another that was either closer to or farther away from the body. For the sentence, “Close the drawer,” the action performed by the participant would be compatible with the sentence if the hand moved away from the body–because the hand usually moves away from the body when closing a drawer–whereas responses toward the participant’s body would be incompatible with the described action. The finding was that action-compatible responses were faster than action-incompatible responses, hence the action-sentence compatibility effect, or ACE.

Aravena et al. [34] looked for neural signatures of the ACE by examining motor and semantic processes indexed by event related potentials (ERPs). They found brain markers of a bidirectional impact of language comprehension and motor processes. Participants were asked to keep their hands in a pre-assigned shape, an open hand or a closed hand, throughout the experiment. Participants then indicated the moment of comprehension of sentences by pressing a button using the shaped hand. Orthogonal to hand-shape, the experimental sentences described actions performed with a closed hand (e.g., “He needed to drive the nail correctly, so Joseph hammered it”), an open hand (e.g., “The show was praiseworthy, so Rocio applauded”), or no hand action (e.g., “After waiting a long time to see his grandmother, Amaro visited her”). Behavioral results showed that participants were quicker to press the response button when the hand-shape required to respond was compatible with the hand-shape implied by the sentence.

In addition, Aravena et al. [34] conducted ERP analyses to investigate the temporal and functional mechanisms of action-sentence comprehension and thus to distinguish among several hypotheses. One hypothesis is that reading a sentence might prime (or more generally, facilitate) a particular hand movement (e.g., a closed hand movement for a sentence about hammering), resulting in faster movement time. A second hypothesis is that preparing to move in a particular fashion (e.g., downward with a closed hand) might speed comprehension of a compatible sentence. Third, both accounts might be correct, resulting in a bidirectional hypothesis: embodied comprehension facilitates movement, and movement facilitates comprehension.

These hypotheses can be disentangled using ERP measures. That is, the bidirectional hypothesis of action-sentence comprehension can be decomposed into two simultaneous effects: motor preparation facilitates language processing (i.e., motor-to-semantics direction), and language processing facilitates activity in movement-related areas (i.e., semantics-to-motor direction). The Aravena et al. [34] ERPs results showed that cortical markers of motor process were affected by sentence meaning, that is, there was evidence for a semantics-to-motor effect. In particular, motor responses elicited a motor potential (MP) before the overt response and this effect was enhanced in the compatible sentence condition. Similarly, in the motor-to-semantics direction, brain markers of comprehension process (e.g., N400-like components) were modulated by motor effects. Thus, the bidirectional hypothesis was supported.

The ACE has also been identified in comprehending sentences containing abstractions, such as time shifts. Sell & Kaschak [35] explored the ways that understanding the abstract concept of time is grounded in the concrete understanding of the space around our bodies. In particular, they investigated if time is organized along the front–back axis (with future events represented as being in front of the body, and past events represented as being behind the body [13], [36]. Participants read sentences involving past or future events and made sensibility judgment responses in one of two ways: (1) moving toward or away from their bodies and (2) pressing the toward or away buttons without moving. Previous work suggests that spatial compatibility effects should be observed, where the future is mapped onto responses away from the body, and the past is mapped onto responses toward the body. These effects were observed, but only when participants were moving to make their responses, and only for larger time shifts (e.g., a month).

### 1.3 ACE in Comprehension of Quantity Information

The results of linguistic analyses suggest that an ACE-like phenomenon occurs while participants are processing sentences including quantity information. This phenomenon is dependent upon the research on the metaphorical representation of “More is up.” Lakoff and Johnson [13] argue that common linguistic expressions (e.g., *My work is piling up; Student enrollment dropped this year*) indicate that speakers of English use a *more is up/less is down* metaphor to structure thought about quantity information. As evidence for this claim, Langston [37] demonstrated that participants were slower to read sentences that violated the *more is up* metaphor (e.g., “He placed Sprite above Coke because it had less caffeine”). Thus, Lakoff and Johnson [13] and Langston [37] both suggest that language users employ the up-down axis when thinking about quantity.

In a series of behavioral studies, the ACE was used to explore the extent to which spatial and motor representations are activated during the comprehension of language about quantity information in both Chinese [38] and English [39]. The goal was to determine which spatial axis (up-down, left-right, or both) would be activated during the comprehension of language about quantity. In Experiment 1 of both papers, participants used response keys aligned on the up-down axis to perform the reading task. In Experiment 2 of both papers, the response keys were aligned on the right-left axis. Participants were asked to read four short stories sentence-by-sentence. Each story included six sentences that contained quantity information (e.g., “More/Less runs were being scored this game”). Participants held down one button to read the sentence (e.g., the top button on the up-down axis) and had to move to press the other button (e.g., the bottom button) to indicate that they finished reading the sentence and were ready for the next one. The results were that for “less” sentences, responses were faster in the down direction, and for “more” sentences, responses were faster to the up direction. When responding was on the left-right axis, there were no significant differences between conditions. These results suggest that quantity and motor compatibility effects are observed when reading sentences about quantity information, and that these effects are observed only on the up-down axis. This interaction between spatial and motor representations is a type of ACE.

Thus, the behavioral studies have demonstrated an ACE in comprehending the abstractions of quantity information in sentences: When processing quantity information such as “more” the participants are quicker in executing upward hand movements. However, the total response time covers two stages, namely, the time spent in comprehending the sentence as well as the time spent in executing the hand movement. Therefore, as in the case of the Aravena study, we need to distinguish among several hypotheses. First, reading a sentence might facilitate a particular hand movement (e.g., upward for a “more” sentence), resulting in faster movement time. Second, preparing to move in a particular default direction (e.g., upward) might speed comprehension of a compatible sentence. Third, both accounts might be correct, resulting in a bidirectional hypothesis: embodied comprehension facilitates movement, and movement facilitates comprehension. Again, similar to Aravena et al., these hypothesis can be disentangled using both behavior and EEG measures as described next.

### 1.4 The Current Study

We asked the participants to hold down a button while reading sentences, some of which ended with the Chinese logogram corresponding to “more and more” or “less and less.” Upon understanding the sentence, the button was released and another button, either in an upward or downward direction, was depressed. Thus, the time between onset of the critical logogram (the last in the sentence) and release of the first button results in a measure of reading (understanding) time (ReT). The participants’ subsequent upward or downward movement to press the other button resulted in a measure of movement time (MoT). MoT provides an index of how comprehension can affect action.

The ReT is not strictly a measure of comprehension time (and thus how action might affect comprehension) because it can be decomposed into the time needed to comprehend the sentence and the time needed to release the key. However, ERPs allow us to reveal the time-locked and response-locked brain-markers. Thus, we can disentangle these two stages and clarify the effects in terms of action-semantics direction.

Semantic processing has been tracked with the N400 component, a large negative deflection in the ERP occurring approximately 400 msec after the presentation of a word. Typically, the N400 is larger when a stimulus is difficult to integrate into a previous semantic context [40]. The N400 effect has been reported for semantic violations in language and for the processing of other meaningful stimuli [41]. Moreover, many researchers have found another negative ERP component functionally like the N400, named N400-like, and these N400-like effects are not restricted to linguistic stimuli [42].

In the current study, the content of the sentences included quantity information intended to activate the “More is up” metaphor. Consequently, the congruent or incongruent conditions were a combination of quantity processing and motor performance (moving up/down response). Because one of the parts of the incongruent condition of this study was not linguistic but a motor response, we expected to find an N400-like modulation.

We presented the Chinese sentence stimuli by rapid serial visual presentation (RSVP) in which a compound character was presented in each time interval (i.e., each presentation consisted of a 1–4 character Chinese word). This presentation mode was used to measure the N400-like component in the ERP analyses. In addition, we measured MP starting 100 msec before the hand was lifted to index any effect of semantics on motor performance.

## Methods

The current project was approved by Institution Review Board of BNU (Beijing Normal University) & ECNU (East China Normal University) (IRB Approval Number is 20100917.) The participants signed their agreements on the informed consent form before their EEG data were collected and were allowed to quit in the middle of experiment at any time without any further explanation. The data were submitted for analysis anonymously. The participants have the right to inquire about their performance, and their data will be saved and only used for research purposes.

### 2.1 Participants

Twenty undergraduate students (10 females) from four universities in Beijing aged 19 to 24 (M = 21.2 years, SD 3.5) participated in this study. They were all native Chinese speakers and right-handed, with normal auditory acuity, normal or corrected-to normal vision, and no reported history of psychiatric or neurological illness. Before the experiment, all participants read and signed an informed consent document. After the experiment, each of the participants received 60 yuan RMB (about $10) as compensation for their participation.

### 2.2 Apparatus

**Figure 1:**
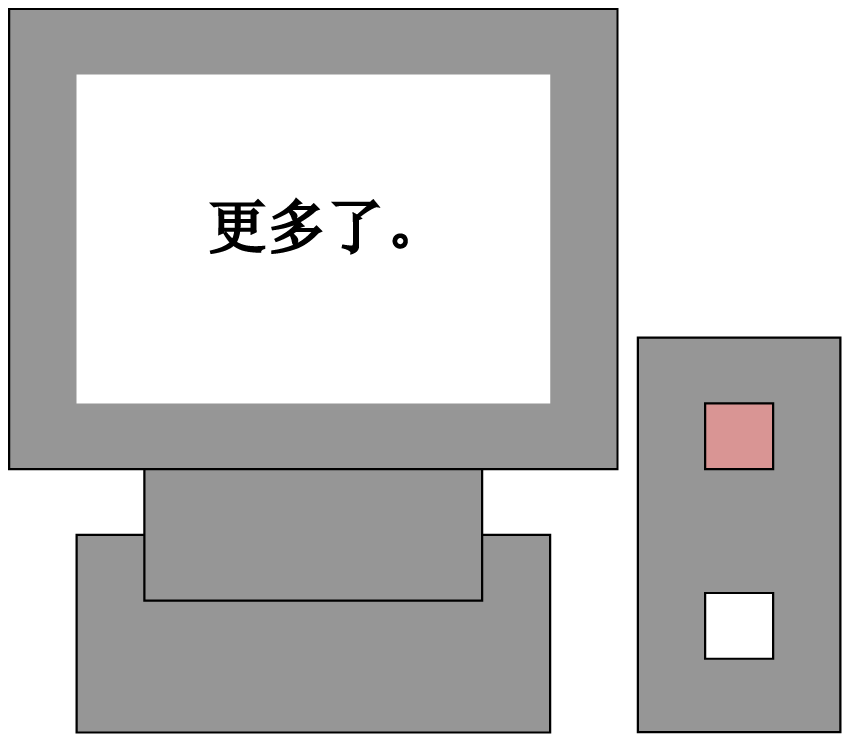
Experimental apparatus and response keyboard.

We designed a special response panel (see Figure 1) that included only two buttons. The button on the upper part of the panel was pink, and the button on the bottom of the panel was white.

### 2.3 Stimuli

Sixteen stories were adapted from the Speer & Zacks [43] reading materials and translated into Chinese (see Example Texts S1 in supporting information). The number of sentences in each story varied from 23 to 27. The number of Chinese words in each sentence varied from 2 to 6. The number of characters in each Chinese word varied from 1 to 4. The stories were homogenous in nature in terms of discourse features, organizational structure, presentation length, as well as the presentation of the target compound characters. Each sentence was presented in 2 to 6 screens (one word on each screen), and the full stop (e.g., “°”) always appeared on the same screen along with the last compound character of each sentence. In each story, there were eight key sentences for which the last compound word was the target character 
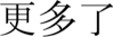
 (more) or 
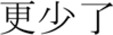
 (less). Four sentences included “
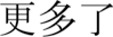
”(more) information, while the other four sentences included “
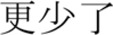
”(less) information. The more or less information was unpredictable from the context of the story. The selection of either more or less information in each story was pseudo-random.

Other than these eight critical sentences in each story, the other sentences did not include any quantity information, so they were all used as filler items. At the end of each story, there were four comprehension questions to encourage the readers to pay attention to the story. The correct answer to two of the questions required inferences based upon the situation of the story. The other two questions probed the quantity information in the story. The data were rejected if the accuracy rate to these questions was below 70%.

Among the sixteen stories, four were used as reading practice materials to help students become familiarized with the experimental procedures and response mode. The other twelve stories were used as the experimental materials. These twelve stories were divided into four blocks of three stories. The presentation order of the three stories in each block was random. The participants changed the direction of the hand movement between blocks, and there was a break between blocks.

### 2.4 Procedure

The experiment was conducted in an electrically- and sound-shielded room under dim lighting condition. The stimuli were programmed using E-prime. The compound characters (i.e., 1–4 character Chinese word) were presented in white on a black background. The participants were comfortably seated behind a desk facing a computer display with a distance ranging from 70 to 100 cm.

**Figure 2:**
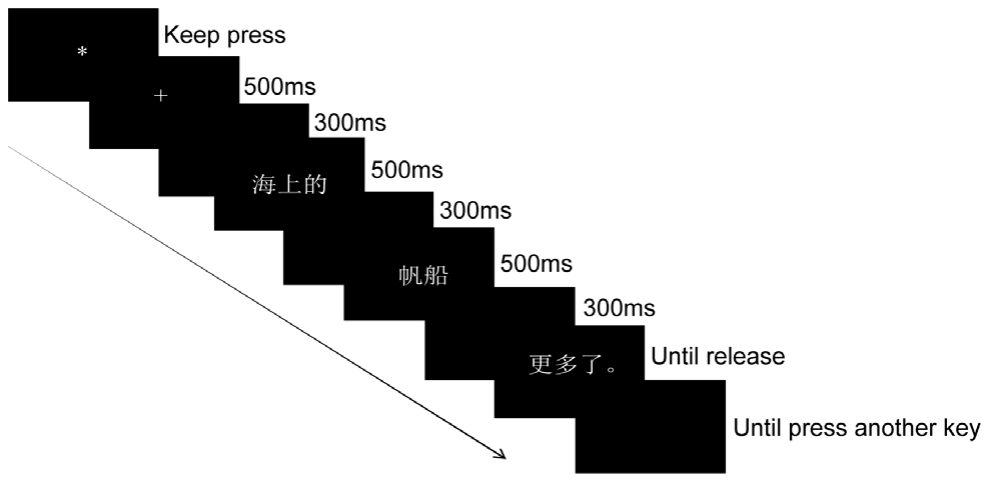
Procedure for story presentation.

Each sentence began with an asterisk, and each compound word stayed on screen for 500 msec and was followed by an inter-stimulus interval of 300 msec. A period occurred at the end of the last compound word of each sentence, indicating the end of each sentence. Participants were engaged in the RSVP reading task (compound word by compound word). In the Upward (U) condition, the participant was required to depress the white (bottom) button throughout the presentation of the sentence, and then to move to the pink button upon seeing the period and comprehending the sentence. The next sentence was initiated by depressing the white button again. The procedure was identical in the Downward (D) condition, except that the pink (upper) button was depressed during presentation of the sentence and the participant moved to the white (bottom) button upon comprehending the sentence. This procedure is illustrated in Figure 2.

The movement direction (U or D) was manipulated within-subjects. To balance the button-press movement among the four reading blocks, the movement patterns were counterbalanced with two orders, UDDU and DUUD, across the four blocks. Before each block of reading, the participants practiced the hand movement by reading one of the four practice stories.

To answer the comprehension questions at the end of each story, participants were required to press “1” for “Yes” and “3” for “No” on the main computer keyboard. The participants received feedback if their answers were incorrect. After reading each story, the participants saw “PAUSE” on the computer screen, indicating that they could rest before reading the next story.

### 2.5 Design

Based upon the More and Less feature of the target sentences and the U or D direction of the action execution, the stimuli were classified into four types: (1) More quantity information+Upward movement (MU), (2) More quantity information+Downward movement (MD), (3) Less quantity information+Upward movement (LU), (4) Less quantity information+Downward movement (LD). As it is assumed that congruent and incongruent movements behave identically independent of the direction of the movement, we compared congruent and incongruent trials within a 2-level factor, i.e., MU and LD comprise the quantity-action congruent condition, whereas MD and LU comprise the quantity-action incongruent condition.

### 2.6 Data Acquisition and Pre-processing

High density EEG was acquired by a Neuroscan ESI-64 system, using a Quik-cap with 64 scalp electrodes placed according to the international 10/10 system and referenced to the left mastoid electrode. Vertical electrooculograms (vEOG) were recorded in a bipolar mode via electrodes positioned above and below the subject's left pupil, and horizontal electrooculograms (hEOG) were recorded from electrodes on the outer canthi of each eye. Scalp-electrode impedances were below 5 KΩ. EEG and EOG were DC continuously sampled at a rate of 1000 Hz/channel on a Synamps 2 system (Neuroscan) with a lowpass of 100 Hz.

The initial EEG data were first offline DC-corrected and re-referenced to the left mastoid electrode. Vertical eyeblink artifact was corrected in the continuous EEG files using the algorithm developed by Semlitsch et al. [44] as implemented by Neuroscan software. The continuous EEG data was divided into epochs spanning from 200 ms pre-stimulus and 1000 msec post-stimulus for stimulus-locked segments, and 800 ms pre-response and 1000 ms post-response for response-locked segments. Epochs were then baseline-corrected relative to the –200 to 0 msec interval before stimulus onset for stimulus-locked segments, and −800 to −600 msec interval before response onset for responses-locked segments. Epochs containing voltage deviations exceeding ±70 µV (stimulus-locked segments) or ±90 µV (response-locked segments) relative to baseline at any of the recording electrodes were rejected in order to exclude artifacts most commonly due to head movements and blinks. ERP waveforms were averaged separately for each electrode for each stimulus type (congruent and incongruent), and then digitally lowpass-filtered at 30 Hz (with 24 dB/octave).

### 2.7 Data Analysis

#### 2.7.1 Behavioral analysis

The behavioral data were analyzed by SPSS 17.0. There were three dependent variables: ReT, MoT, and the total. Trials were rejected if the total was not between 200 and 3000 msec. The statistical significance level was 0.05.

#### 2.7.2 ERP analysis

ERP analyses focused on stimulus-locked activity elicited by individual stimuli presented in the end of the key sentence (i.e., the key word) and response-locked activity elicited by individual responses that occurred after understanding every key sentence (i.e., congruent and incongruent sentences).

#### 2.7.3 N400-like

For stimulus-locked activity, the N400-like component was found over frontal and central electrode sites (at approximate FZ, FCZ and CZ positions) between 400 and 600 msec, observed from initial visual inspection of grand mean waveforms. In previous studies, the classical N400-effect is reflected in a stronger negative waveform for semantically incongruent compared to congruent sentence-final words and is found maximal above central-parietal areas around 400 msec after word onset [40], [42], [45]. In addition to the classical N400-effect, several studies have reported an anterior N400-effect with a stronger negative amplitude for the processing of concrete compared to abstract words (i.e. the N400-concreteness effect; [46]–[47], which is comparable to the anterior N400-effect found in association with the processing of picture stimuli [48]–[51]. In this experiment, the frontal and central electrode sites and 450–550 ms time window were selected for statistical analysis of the N400-like component.

From initial visual inspection, the difference in N400-like amplitude between congruent and incongruent stimulus types lateralized in left and middle regions. In order to discuss the effect of lateralization and electrode location, the anterior brain area was divided into 9 sub-areas, 3 (frontal, fronto-central and central areas on the front-back axis)×3 (left, middle and right areas on the left-right axis). The corresponding electrodes were F5/FZ/F6, FC5/FCZ/FC6 and C5/CZ/C6. This analysis is consistent with previous reports for maximal location of motor responses [52] and the N400-like component [45].

#### 2.7.4 MP

For response-locked activity, the mean negativity amplitude of motor potential (MP) was measured between 100 msec prior to motor onset and motor onset, according to the time window for MP selected in Slobounov et al. [50](2002). MP reflected the cortical activation associated with later stages for motor preparation [54]. From the initial visual inspection of grand mean waveforms, the MP components in this experiment were mainly distributed over frontal and central electrode sites (at approximate FZ, FCZ and CZ positions), which was in accordance with the central areas reported in previous studies [52]–[53], [55]–[56].

In analyzing MP, the frontal and central brain area was divided into 12 sub-areas, including frontal-left, frontal-middle, frontal-right; fronto-central-left, fronto-central-middle, fronto-central-right; central-left, central-middle, central-right; centro-parietal-left, centro-parietal-middle, centro-parietal-right. The corresponding electrodes were F3/FZ/F4 for frontal, FC3/FCZ/FC4 for fronto-central, C3/CZ/C4 for central, CP3/CPZ/CP4 for centro-parietal area.

#### 2.7.5 Statistical analysis

A repeated-measures analysis of variance (ANOVA) was performed on the mean amplitude of the ERPs to the critical words for each of the 9 electrode sites separately in the N400-like (450–550 msec) and MP (−100–0 msec) time windows. The factors in the ANOVA were Type (congruent, incongruent)×Electrode location (frontal, fronto-central, central)×Lateralization (left, middle, right).

To decrease the experimentwise error rate due to the repeated-measures design involving multiple dependent variables, a Greenhouse and Geisser adjustment of the degrees of freedom was performed. This correction was applied when the violations of sphericity for significant effects occurred in the analysis of variance with two or more degrees of freedom. Post hoc testing was conducted only when preceded by a significant analysis of variance effects using Bonferroni adjustment. For all measures, statistical significance was taken as *p*<0.05.

## Results

### 3.1 Behavioral Results

We analyzed Total response time, ReT, and MoT. The most relevant means are presented in Table 1. Total response time was measured from the onset of the target compound characters to the time when participants pressed the second button. A paired-sample t-test showed that total response time in the incongruent condition was significantly longer than that in the congruent condition (*t*_(19)_ = −3.280, *p* = 0.004). For ReT, the mean in the congruent condition was shorter than that in incongruent condition (*t*_(19)_ = −2.112, *p* = 0.048). Similarly, for the MoT the mean in the congruent condition was shorter than that in incongruent (*t*_(19)_ = −2.166, *p* = 0.043). These results demonstrate the behavioral ACE.

**Table 1:** Total, ReT, and MoT for congruent and incongruent conditions (msec).

	ReT		MoT		Total	
	RT	SD	RT	SD	RT	SD
Congruent	589.68	89.68	502.90	104.30	1092.58	168.76
Incongruent	600.86	91.64	513.92	111.21	1114.78	180.65

### 3.2 ERP Results

#### 3.2.1 N400-like

**Figure 3:**
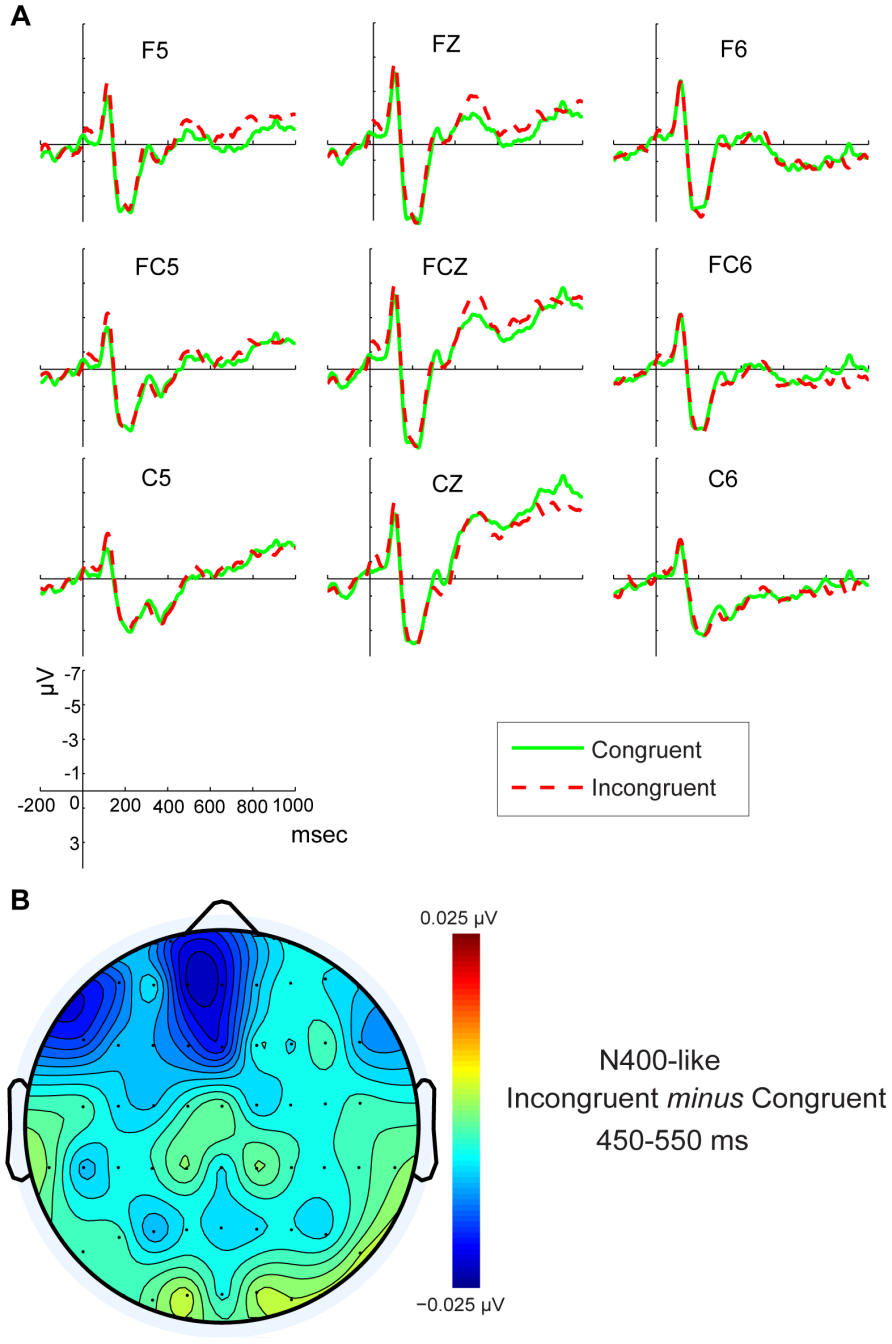
ERP waveforms and topographic maps of N400-like component. A) Grand-averaged stimulus-locked ERP waveforms for congruent and incongruent conditions for frontal central regions.B) Topographic maps of N400-like component after subtraction of the Congruent condition from the Incongruent condition.

For stimulus-locked ERPs, the stimuli exhibited an N400-like component in the frontal and central region in 450 to 550 msec time window. The grand average N400-like waveforms at the 9 electrode sites (F5, FZ, F6, FC5, FCZ, FC6, C5, CZ, C6) for congruent and incongruent stimuli type are shown in Figure 3.

Consistent with previous research [34], both visually and statistically, the larger amplitude of the N400-like component was observed under Incongruent than Congruent conditions, which is considered an ACE effect. In our findings, the ACE effect was observed at most of the anterior regions. The ANOVA yielded a significant effect of Type (F _(1, 20) = _5.159, *p* = 0.034). The mean amplitude for Incongruent trials (M = −1.85 µV, SD = 0.55) was more negative than for Congruent trials (M = −1.13 µV, SD = 0.56), suggesting an ACE which discriminated incongruent stimuli from congruent. A significant effect of Type×Electrode location (F _(2, 40)_ = 7.06, *p* = 0.004) was also found. Post hoc comparisons performed on this interaction effect showed that Incongruent stimuli elicited enhanced N400-like amplitudes compared with Congruent at frontal (F _(1, 20)_ = 10.459, *p* = 0.004) and fronto-central areas (F _(1, 20)_ = 5.561, *p* = 0.029), but not central areas (F _(1, 20)_ = 0.878, *p* = 0.360). Most important, the three-factor interaction including Type×Electrode location×Lateralization (F _(4, 80)_ = 3.884, *p* = 0.026) was significant. Post hoc comparisons showed that only at F5 (F _(1, 20)_ = 5.971, *p* = 0.024), FC5 (F _(1, 20)_ = 3.207, *p* = 0.088), FZ (F _(1, 20)_ = 16.023, *p* = 0.001) and FCZ (F _(1, 20)_ = 11.075, *p* = 0.003) electrode sites was the N400-like mean amplitudes elicited by Incongruent stimuli significantly greater than for Congruent stimuli, indicating a left-middle anterior distribution of the ACE. These results demonstrate an electrophysiological ACE and indicate and effect of motor preparation on semantic analysis.

#### 3.2.2 MP

**Figure 4:**
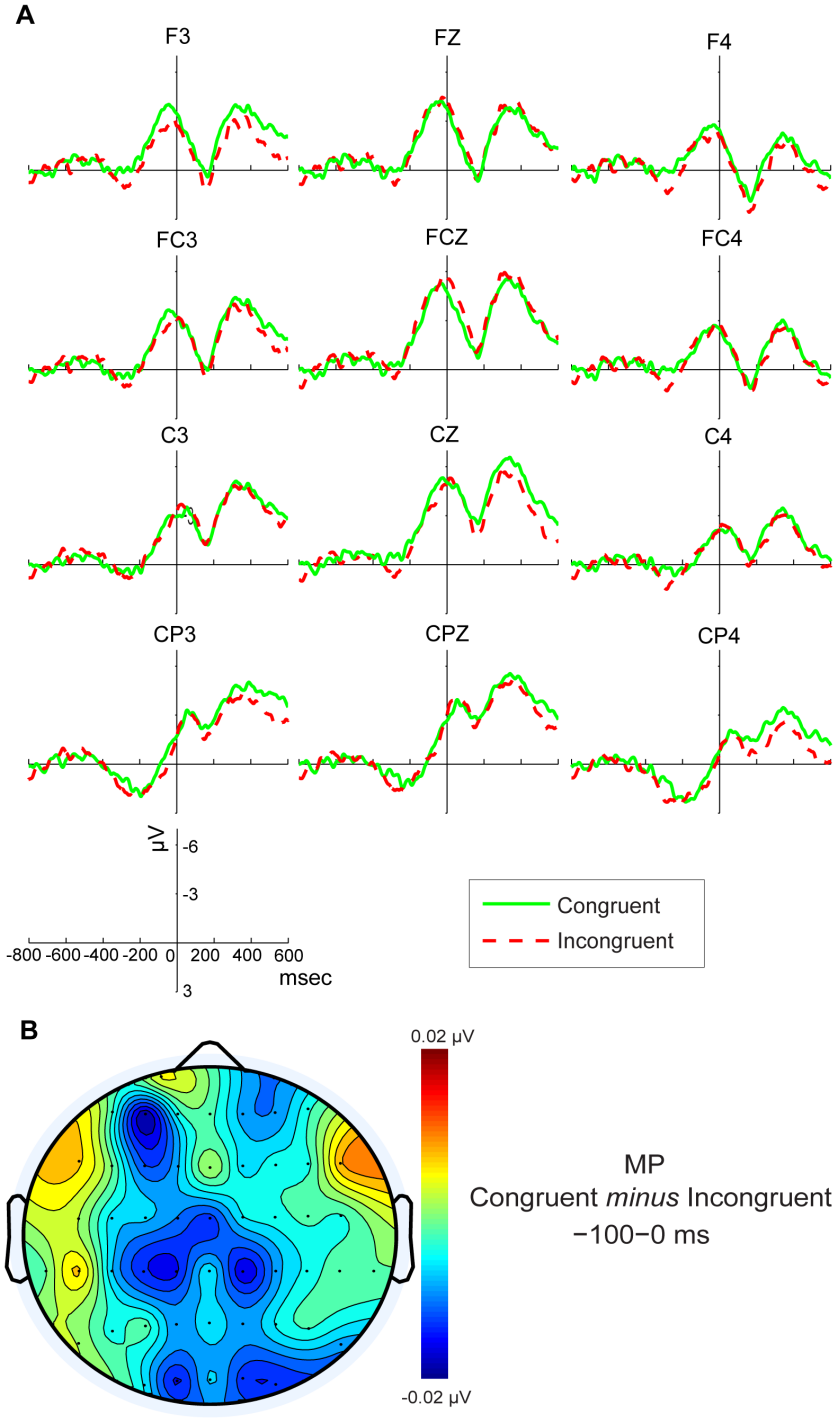
ERP waveforms and topographic maps of MP component. A) Grand-averaged response-locked ERP waveforms for congruent and incongruent conditions for frontal and central regions.B) Topographic map of the MP component after subtraction of the Incongruent condition from the Congruent condition.

The response-locked ERPs are locked to the release of the first button, and the MP component is measured in the −100 to 0 msec time window (MP) at the frontal and central region. Figure 4 illustrated the grand average MP waveforms at the 12 electrode sites (F3, FZ, F4, FC3, FCZ, FC4, C3, CZ, C4, CP3, CPZ, CP4) for congruent and incongruent stimuli type. From visual inspection, Congruent stimuli elicited enhanced MP negative amplitudes compared with Incongruent stimuli at central regions, which was consistent with previous research. The ANOVA yielded a significant effect of Type×Electrode location×Lateralization (F _(6, 114)_ = 2.538, *p* = 0.047). Post hoc comparisons showed that only at F3 (F _(1, 19)_ = 4.578, *p* = 0.046), CZ (F _(1, 19)_ = 4.444, *p* = 0.049) and CP3 (F _(1, 19)_ = 5.533, *p* = 0.030) electrode sites was MP mean amplitude elicited by Congruent stimuli significantly greater than for Incongruent stimuli. These data indicate a left frontal and central distribution of the ACE due to an effect of the sentence semantics on motor activity.

## Discussion

The goal of our experiment was to determine if there is a correspondence between semantic processing of a sentence containing quantity information and motor activity when that motor activity is congruent or incongruent with the quantity information. The behavioral evidence consisted primarily of the time needed to understand the information and make a response. The times were faster in the Congruent condition compared to the Incongruent condition: An ACE. Specifically, in the Congruent condition, the total response time, release time (ReT) and movement time (MoT) were all significantly shorter than those in the Incongruent condition.

The ERP results included both stimulus-locked ERPs and response-locked ERPs. In stimulus-locked ERPs, we found an N400-like component in the frontal central region in the 450 to 550 msec time window, with the mean amplitude of the Incongruent condition more negative than that of Congruent condition. A larger N400-like component in the incongruent condition suggests a difficulty in integration with a semantic context. Thus, this compatibility effect demonstrates how motor activation (moving upward or downward) interacts with semantic analysis.

It is worth commenting on the localization of the N400-like effect. Previous studies on language processing found that the scalp distribution of the N400-like component is mainly localized at posterior association cortices in the left hemisphere. In contrast, our findings indicate that the stronger N400-like component appeared over the mesio-frontal and frontal-lateral cortex for the incongruent trials. This location suggests that some action monitoring mechanism might be involved when the motor system is engaged in language comprehension, as anterior cingulate (ACC) appears to play a crucial role in initiation, motivation, and goal-directed behaviors [57]–[59]. As we discuss below, the motor system implements forward models that predict the sensory consequences of actions. Checking these predictions might generate the frontal signal. In any event, the issue of localization of the N400-like component when the motor system is involved in language comprehension deserves further research.

In response-locked ERPs, we identified the MP before the first release of a button indicating that the sentence was understood and initiating movement upward or downward. The higher MP amplitudes (i.e., mean amplitude of the negative brainwaves) for the Congruent condition suggest that sentence content facilitates the precision and speed of a Congruent action. This finding constitutes evidence that semantic analysis affects responding.

Earlier studies have suggested the involvement of the motor system in language processing during the first 200 ms of stimulus presentation (e.g., [60]–[61]). However, we did not observe evidence for motor system involvement in this early window. One reason for this difference might be noise [34], particularly given the relatively few observations in our analysis. Another possibility is that most studies showing the early effect used verbs to reveal the relationship between language and action, whereas our study used abstract quantifiers. The quantifier may need to be integrated with the context before any motor simulation becomes relevant, and this integration could take several hundred milleseconds [29].

Overall, the data are clear in showing signs of motor system activity during the comprehension of abstract language. Next, we consider what that activity means by answering four questions. (1) Why is there any relation between the motor system and language comprehension? (2) Is the relation causal, that is, does motor activity play a causal role in language comprehension? (3) Is the relation one of constituency? In other words, is that motor activity the comprehension itself? (4) How can an abstract idea be based on motor activity?

### 4.1 Why the Motor System Plays a Role in Language Comprehension

As noted in the introduction, language and sensorimotor activity have traditionally been treated as separate cognitive processes. Nonetheless, both behavioral and neurophysiological research over the last two decades have shown strong connections between language and action. Our first question is why that should be. One answer is given by the simulation approach to language comprehension. Namely, language comprehension results when the linguistic symbols drive activity in sensorimotor and emotional systems into states that are homologous to the states engendered by literal experience in the situation described by the language. Thus, reading about a sad situations drives emotional systems into states homologous to those when one is literally sad [62]; reading about visual movement drives visual perceptual systems into states homologous to those when one literally observes visual motion [63]; and reading about action drives action systems into states homologous to those when literally acting [14].

But, there is also a deeper answer to the “why” question. Namely, there appears to be a strong evolutionary connection between language and the motor system. This connection may reflect how the mirror neuron system plays a role in gestural communication and vocal communication [64].Compelling evidence for this connection comes from studies reported by Gentilucci and colleagues (e.g., [65]–[66]), who demonstrate interference between manual and oral activity.

### 4.2 The Causal Role of the Motor System in Language Comprehension

There are two alternatives to the simulation answer. The first is that motor activity is simply an epiphenomenon. The second is that motor activity reflects a process of motor imagery after comprehension, but in fact that motor activity has little to do with the process of comprehension itself. These alternatives lead us to the second question: Is the relation between motor activity and language comprehension causal? The data from the current research (as well as other data) speak against the motor imagery alternative. The reason is that the EEG signal indicates motor system activity shortly after presentation of the key words, perhaps too soon to reflect a conscious, imaginal process. Pulvermueller (e.g., [67]) comes to the same conclusion for listening to words: activity in the motor system occurs within about 20 msec of peak activity in auditory cortex, which is too short of a time lag to be caused by post-comprehension imaginal processes.

There is also very strong experimental data indicating a causal relation between language and motor activity. For example, Pulvermüller, Hauk, Nikulin, and Ilmoniemi [68] demonstrated that a double TMS pulse to the motor cortex facilitates the naming of action words. And Glenberg et al. [24] were able to demonstrate a causal link behaviorally. They had people repeatedly move objects (cannellini beans) towards themselves or away from themselves. The goal was to differentially adapt the motor system that controls one or the other of these actions. After the adaptation, participants read sentences that described transfer of objects toward or away from the participant. Time to comprehend these sentences depended on both the direction of adaptation and the direction of transfer described in the sentence: After participants adapted the motor system in the toward direction, they were slower to read sentences describing transfer toward them, and the complementary finding was observed after adapting in the away direction. [Why should motor activity slow sentence comprehension? One explanation is that the motor activity fatigued the neural process used in controlling a particular action. A different alternative is that repeatedly moving the beans specialized the motor system for the parameters (e.g., extent, velocity, hand shape) of the adapted movement. Then, when the sentence required a simulation using different movement parameters, the simulation needed to adjust those adapted circuits.].

Of particular interest in the context of the current data, Glenberg et al found the adaptation effect both for sentences describing the transfer of concrete objects (e.g., “Art hands you the pencil”) and the transfer of abstract objects (e.g., “Anna delegates the responsibilities to you”). Thus, we can conclude that the motor system can play a causal role in language comprehension.

### 4.3 The Constitutive Role of Motor Activity in Language Comprehension

The third question is how we should think about the causal connection between language comprehension and action systems. It may be that simulation cannot proceed without the motor system, for example, that the motor system energizes simulation, but that the motor activity is not central to comprehension. The constituency claim is stronger than this causal claim, however. The constituency claim is that the activity in the motor system is itself a part of the understanding.

The case for constituency is difficult to make experimentally because experiments are logically designed to test for causation, not constituency. Thus, the case for constituency must rest on the weight and diversity of the evidence, as well as the reasonableness of alternatives. The data from the current research play a particularly strong role in making the case for constituency. The reason is that the current data demonstrate a bi-directional relation between language and the motor system: The N400-like component demonstrates an effect of required movement on the process of sentence comprehension, and the MP effect demonstrates an effect of the unfolding sentence comprehension on preparation for movement. These effects could be relatively independent forms of priming in which the motor system primes the language system, and the language system primes the motor system. However, the constituency claim is simpler: Rather than two forms of priming, because motor system activity is itself the understanding (or a constituent of the understanding), any relevant change in motor activity changes the understanding and any relevant change in understanding changes the motor activity.

### 4.4 The Motor System and Abstract Language: Forward Models

The fourth question is why any of this should matter for the understanding of abstract information: On first blush, abstract information seems to have little to do with motor activity. We suggest three interrelated answers. The first answer is based on Lakoff and Johnson [13]. As discussed in the introduction, they propose that many abstract concepts are understood through conceptual metaphor, such as “More is up.” But one could also ask about the neural basis for understanding this metaphor, and that is where we get to the motor system. In fact, more is often up because motor activity is used in the stacking of objects that creates a growing pile. In other words, the understanding of “more is up” is grounded in a motor simulation of stacking.

A second answer comes from noting that many abstract terms are names for processes [19]. For example, the concept “cause” can be simulated as a motor process of pushing or a pulling [69]. The term “democracy” refers to a temporally and spatially extended set of processes involving communication, voting, and so on. Thus, “democracy” can be simulated (in part) as motor processes involved in voting and counting votes. In brief, the simulation underlying abstract terms often involves motor processes (but see [23] for evidence that abstract terms are also partially grounded in emotion).

The third and most basic answer derives from the nature of motor control, namely, that it is a system that depends on prediction (e.g., [31], [33]). When planning and initiating action, the motor system sends the motor commands both to the body and to a forward model. The forward model is used to predict the sensory consequences of the intended action, that is, what a successful action will feel like and how the world will change as a result of that action. These predictions can be used to correct movement parameters on the fly, and comparing the predictions to actual sensory feedback determines if the movement was successful. Schubotz and colleagues (e.g., [70]) have documented motor system activity in a variety of motor and (seemingly) non-motor prediction tasks. It has also been suggested (e.g, [71]–[72]) that the forward model is the basis of imagery. That is, when actual movement is suppressed, but the forward model predicts the sensory consequences of the movement, those predictions give rise to the experience of images.

Language processing also involves prediction. In conversation, we predict each other’s words, thoughts, accents, and timing. In fact, both Glenberg and Gallese, [73]and Pickering and Garrod [32] independently invoke forward models and the motor system to account for various aspects of prediction in language. Glenberg and Gallese go further and speculate that language evolved by using the hierarchical control structures of the motor system, including forward models, both for meaning and syntax. In support of these proposals, Lesage, Morgan, Olson, Meyer, & Miall [74] demonstrated how cerebellar rTMS disrupts predictive language processing, presumably by disrupting the operation of forward models.

Different forward models enable the same movement (e.g., raising the arm) to be associated with different goal-directed actions and their effects. For example, raising the arm may be part of a goal-directed action to reach a cup on a high shelf. In this context, the forward model associated with reaching a cup predicts the feeling at the shoulder and hand and the sight of the hand above the head. In a different context, for example stacking blocks, raising the arm allows another block to be stacked on a pile. In this context, the forward model associated with stacking predicts a higher stack with more objects. It is this forward model that underlies the conceptual metaphor “more is up.” And, in the context of a social situation, raising the arm may be a signal of status or power [75]. Here, the forward model produces the prediction that when the arm is raised, others will be obedient. Thus, the same physical action, raising the arm, is understood to have three different meanings by virtue of the predictions that accompany the action.

When different linguistic contexts (e.g., “more and more” or “threat”) invoke similar motor behavior but different forward models, the predictions from the forward models constitute different understandings of the language. That is, an abstract linguistic context invokes a simulation in the motor system; the simulation includes a forward model that makes predictions as to how the motor activity will affect both the body and the world. These predictions underlie the relation between the motor system and abstract language.

Thus, we converge on two general, albeit highly speculative, conclusions. The first is in regard to just what it means to be an abstract concept. The dictionary definition of “abstract” is often a variant of “not directly sensed using sight, audition, smell, taste, or touch.” Such a definition is inadequate at least in part because it disregards interoception and proprioception. A second definition, related to Lakoff and Johnson [13], is that abstract concepts are those understood by metaphorical extension of more concrete concepts. Whereas this is an advance, it does not strongly relate to brain processes.

We propose instead (and related to [19]) that abstract concepts are those that are mainly grounded in the process of prediction, rather than grounded in particular sensorimotor simulations, and prediction is a function of forward models implemented in the motor system. Thus, “banana” is predominantly a concrete concept in that its meaning is grounded in a simulation involving vision (for shape and color), taste, and the motor system (for peeling and eating). However, one can argue that part of the meaning of “banana” is the prediction that its consumption will satisfy a craving or reduce hunger, and thus the concept has some abstract components as well.

In contrast, a concept such as “love” will have some concrete components, e.g., the interoceptive simulations associated with love (see, [23]), but most of its meaning may derive from predictions of what follows from the proposition that X loves Y. Similarly, the concept “democracy” will have some concrete components (e.g., the simulation of voting), but the brunt of its meaning stems from predictions of what follows from a process being democratic (e.g., orderly turnover of governments, concern for human rights, tolerance of dissent; of course all of these relatively abstract notions need to be fleshed out in an embodied system, but doing so here would take us too far afield). Finally, a quantifier such as “more and more” will be grounded in both concrete simulations involving motor activity (e.g., making a pile), but also it will be grounded in predictions regarding what follows from eating more and more bananas, or loving someone more and more.

The second general conclusion is that given this approach to abstract linguistic concepts, the motor system, by virtue of its role in prediction, plays a central role in the understanding of all abstract language. Our data support this conclusion in a particular context. The extent to which this conclusion applies generally remains to be determined.

## Supporting Information

Example Texts S1 **Two texts used in the experiment and approximate English translations.**
(DOC)
