# Supplementary material for: The Motor System Contributes to Comprehension of Abstract Language
Source: PLoS One. 2013 Sep 26;8(9):e75183. doi: 10.1371/journal.pone.0075183 (PMC3784420; doi:10.1371/journal.pone.0075183)
Supplement: Example Texts S1 — Two texts used in the experiment and approximate English translations. (DOC) [file pone.0075183.s001.doc]

Supplementary Material: Two texts used in the experiment and approximate English translations. ^【1】^

| 故事一 | Story 1 [Translated Version] |
| --- | --- |
| 小强/刚上/大一。/ | XiaoQiang/is/a freshman. |
| 今天是/他的/生日。/ | Today is/his/birthday. |
| **送他/礼物的/朋友/更少了。/** | **(The number of ) friends/who gave him/gifts (becomes)/less and less [more and more]^【2】^.** |
| 他收到/一台/崭新的/电脑。/ | He receives/a set of/brand-new/computer. |
| 他打开/箱子/开始/组装/电脑。/ | He opens/the box/to start/to assemble/the computer. |
| **组装/电脑的/零件/更少了。/** | **(The number of) the parts he used/to assemble PC (becomes)/less and less [more and more].** |
| 小强/看了一下/说明书。/ | XiaoQiang/takes a look at/the instruction. |
| **他要做的/事情/更多了。/** | **(The number of )Things/he needs to do (becomes)/more and more [less and less].** |
| 到了/安装/驱动/程序的/时候了。/ | It is time/he should install/ the program of driver. |
| 他很高兴/电脑/主机上/有DVD/刻录机。/ | He is very excited that/there is a DVD/recorder/in his computer. |
| **到他/寝室/串门的/朋友更少了。/** | **(The number of)Friends/dropped by/his room (becomes)/less and less [more and more].** |
| 小强把/显示器/放在/桌子上。/ | XiaoQiang/places/the monitor/on the desk. |
| 他把/打印机/与主机/连接好。/ | He/connects/his computer/with the printer. |
| **电脑/播放的/音乐/更多了。/** | **(The number of pieces) PC/played/music (becomes)/more and more [less and less].** |
| 小强/想要/休息/一下。/ | XiaoQiang/would like to/take/a break. |
| **他想到/要做的事情/更少了。/** | **(The number of) Things/he planned to do (becomes)/less and less [more and more].** |
| 他吃了/一块糖。/ | He/eats/a piece of candy. |
| **人们/在宿舍里/养的宠物/更多了。/** | **(The number of) People/raised pet/in the dorm (becomes)/more and more [less and less].** |
| 小强/快要/安装完/电脑了。/ | XiaoQiang/is about/to complete/the task. |
| 他整理了/一下/乱糟糟的/东西。/ | He puts together/the big mess/of things. |
| 现在/可以/测试/电脑了。/ | Now/he can/test/his computer. |
| **室友们/乱扔的书/更多了。/** | **(The number of) His roommates/threw here and there/books (becomes)/more and more [less and less].** |
| 小强/想给/朋友/打个/电话。/ | XiaoQiang/decides/to make a/phone call/to his friends. |
| 感谢他/送来/电脑/作礼物。/ | He wants/to thank him/to send him/a computer/as a gift. |
| 小强收到的生日礼物更多了吗？ | Does XiaoQiang receive more and more gifts from his friends? |
| 小强可以用电脑刻录DVD光盘吗？ | Can XiaoQiang use the computer to burn DVD? |
| 小强的住处活动空间更少了吗？ | Is the room used for activity in XiaoQiang’s building is less and less? |
| 小强装电脑感到累了吗？ | Does XiaoQiang feel tired while assembling the computer? |

^[1]^We created one version of each text by randomly selecting the use of “More” or “Less” for each sentence; the second version reversed the selection. Half of the participants read Version 1, and the other half read Version 2. There are four comprehension questions in each story. Two of the questions are about quantity information. The other two questions query inferences based upon the context of the story.

^[2]^Although this key sentence translated into English follows the syntactic pattern of “The number of … is more and more/less or less,” the actual Mandarin sentences do not contain “**the number of**” and “**becomes.**” Like other sentences in the story, the syntactic structure of the key sentence follows the pattern of “S+V” in which the last word “more and more” or “less and less”, the quantity information, is used as a simple predicate, which is pretty typical in Mandarin. Therefore, this quantity information is implicit and abstract in the semantic sense.

| **故事2** | **Story 2 [Translated Version]** |
| --- | --- |
| 小丽/在机场/侯机。/ | XiaoLi/is waiting for her flight/at the airport. |
| 她/准备/坐飞机/去看望/姐姐。/ | She/is ready/to fly/to visit/her sister. |
| 她好久/没有/见到/姐姐了。/ | She/ has not/met her sister/ for a long time |
| **带着/孩子/旅游/的人/更多了。/** | **(The number of) passengers/ taking/ children to travel (becomes)/more and more [less and less].** |
| 她低头/扫了一眼/机票。/ | She looks down/to glance at/the flight ticket. |
| 她发现/她的/座位是/第七排。/ | She finds that/her/seat is/at the seventh row. |
| 七/是她的/幸运/数字。/ | Seven/is her/lucky/number. |
| **这个机场/飞机/晚点的/情况/更多了。/** | **(The frequency of) the flights/delay/at this airport (becomes)/more and more[less and less].** |
| 小丽/想去/吃点/东西。/ | XiaoLi/would like to/eat/something. |
| **机场里/她喜欢的/食物/更少了。/** | **At the airport/(the number of) of food/ she liked (becomes)/less and less [more and more].** |
| 她拿出/一本小说/读起来。/ | She/brings out/a novel/to read. |
| **她看书/的兴趣/更多了。/** | **(The amount of) her interest/in reading (becomes)/more and more [less and less].** |
| 小丽/拿起/她的/钱包。/ | XiaoLi/takes out/her/purse. |
| 她想/确认/是否带了/护照。/ | She would like to/check/whether she has brought/her passport. |
| **来打扰/她的/电话/更少了。/** | **(The number of) phone call/disturbed her (becomes)/less and less [more and more]**. |
| 她想要/读一些/轻松点的/文章。/ | She would like to/read some/interesting/articles. |
| **窗外/起飞的/飞机/更少了。/** | **Out of the window/(the number of)flight/taking off (becomes)/less and less [more and more].** |
| 她/看了一下/手表。/ | She/takes a look at/her watch. |
| 她查看/手机的/语音/信箱。/ | She/checks/the voicemail/in her cell phone. |
| **在她/旅游时/来的电话/更少了。/** | **(The number of ) incoming phones/during her traveling (becomes)/less and less [more and more].** |
| 她看到/人们/都从/座位上/站起来了。/ | She notices/that all of the people/stand up/from their seats. |
| 小丽/取出/她的/机票。/ | XiaoLi/takes out/her/light ticket. |
| **四处/走动的/的人/更多了。/** | **(The number of) people/walking/up and down (becomes)/more and more [less and less].** |
| 她终于/起程了。/ | She finally/takes off. |
| 小丽和姐姐不在同一个城市？ | Do XiaoLi and her sister live in the same city? |
| 在候机中，小丽感到饥饿了吗？ | While waiting for the flight, does XiaoLi feel hungry? |
| 飞机晚点的情况更少了吗？ | Are there fewer flight delays recently? |
| 小丽旅行中手机语音信箱的来电更多了吗？ | During her travel, are there more incoming voicemails in her cell phone? |
